# Ankyrin-B Coordinates the Na/K ATPase, Na/Ca Exchanger, and InsP_3_ Receptor in a Cardiac T-Tubule/SR Microdomain

**DOI:** 10.1371/journal.pbio.0030423

**Published:** 2005-11-29

**Authors:** Peter J Mohler, Jonathan Q Davis, Vann Bennett

**Affiliations:** **1**Department of Pathology, Vanderbilt University, Nashville, Tennessee, United States of America,; **2**Howard Hughes Medical Institute and Departments of Cell Biology, Biochemistry, and Neurosciences, Duke University Medical Center, Durham, North Carolina, United States of America; University of Utah Health Sciences CenterUnited States of America

## Abstract

We report identification of an ankyrin-B-based macromolecular complex of Na/K ATPase (alpha 1 and alpha 2 isoforms), Na/Ca exchanger 1, and InsP_3_ receptor that is localized in cardiomyocyte T-tubules in discrete microdomains distinct from classic dihydropyridine receptor/ryanodine receptor “dyads.” E1425G mutation of ankyrin-B, which causes human cardiac arrhythmia, also blocks binding of ankyrin-B to all three components of the complex. The ankyrin-B complex is markedly reduced in adult ankyrin-B^+/−^ cardiomyocytes, which may explain elevated [Ca^2+^]_i_ transients in these cells. Thus, loss of the ankyrin-B complex provides a molecular basis for cardiac arrhythmia in humans and mice. T-tubule-associated ankyrin-B, Na/Ca exchanger, and Na/K ATPase are not present in skeletal muscle, where ankyrin-B is expressed at 10-fold lower levels than in heart. Ankyrin-B also is not abundantly expressed in smooth muscle. We propose that the ankyrin-B-based complex is a specialized adaptation of cardiomyocytes with a role for cytosolic Ca^2+^ modulation.

## Abstract 2

The authors describe an ankyrin-B-based macromolecular complex localized in cardiomyocyte T-tubules, which the authors propose is a specialized adaptation of these cells with a role for cytosolic Ca^2+^ modulation.

## Introduction

Defects in Ca^2+^ homeostasis underlie major diseases of the heart including congestive heart failure, cardiac hypertrophy, and fatal cardiac arrhythmias [1,2]. Ca^2+^ ions enter cardiomyocytes through voltage-sensitive Ca^2+^ channels (dihydropyridine receptor [DHPR]) located in invaginations of the plasma membrane known as transverse tubules (T-tubules). DHPR is localized in a microdomain of the T-tubule that is synapsed with sites in the sarcoplasmic reticulum (SR) that are enriched in Ca^2+^-release channels (ryanodine receptor [RyR]; [3,4]). Ca^2+^ that enters through DHPR must be balanced in each contraction cycle (~100 ms in mouse) by Ca^2+^ export. Ca^2+^ export is accomplished primarily by the Na/Ca exchanger 1 (NCX1), which is driven by the transmembrane Na^+^ gradient provided by the Na/K ATPase (NKA) [5]. The requirement for rapid export of Ca^2+^ is a specialized feature of heart that is not present in skeletal muscle, where DHPR directly activates RyR without Ca^2+^ import.

Ca^2+^ export has historically been an important therapeutic target in the management of heart failure. Cardiac glycosides increase [Ca^2+^]_i_ by inhibiting NKA, thus elevating [Na^+^]_i_ and indirectly inhibiting Ca^2+^ efflux through NCX1 [6]. Several considerations suggest that NCX1 and NKA operate together in diffusion-limited physiological spaces. Cardiac glycosides do not elevate averaged cytoplasmic [Na^+^] to levels sufficient to inhibit NCX1, suggesting NKA and NCX are coupled [6,7]. Arrhythmia caused by cardiac glycoside toxicity is believed to result from a transient inward current carried by NCX1 [8]. However, NCX1 would not be expected to generate inward flux of Na^+^ if averaged [Na^+^] was elevated sufficiently to inhibit Ca^+2^ efflux. Numerous studies support a tight functional coupling between NCX1, NKA, and intracellular Ca^2+^ stores in heart [9–13]. However, while co-localization between NCX1, NKA, and intracellular Ca^2+^ stores is described in smooth muscle [14], the relative localizations of these proteins in heart are undefined. Additionally, no biochemical evidence exists for a direct link between NKA, NCX1, and other SR proteins in cardiomyocytes.

Ankyrin-B is a multivalent adapter present in cardiomyocytes that binds individually to NCX1, NKA, and inositol 1,4,5-trisphosphate receptors (InsP_3_Rs), and potentially could play a role in functional coupling of these proteins [15–17]. Loss-of-function mutations in ankyrin-B cause a dominantly inherited human cardiac arrhythmia syndrome associated with sudden cardiac death [15,18]. Mice heterozygous for a null mutation in ankyrin-B (ankyrin-B^+/−^ mice) are haploinsufficient and have a similar cardiac phenotype as humans heterozygous for loss-of-function mutations of ankyrin-B [15,18]. Adult ankyrin-B^+/−^ cardiomyocytes exhibit elevated [Ca^2+^]_i_ transients and, in the presence of beta-catecholamines, exhibit delayed and early afterdepolarization events and extrasystoles [15]. In contrast, action potential duration, inward Ca^2+^ current, and diastolic [Ca^2+^] are normal in ankyrin-B^+/−^ cardiomyocytes [15].

We report here that NKA (alpha 1 and alpha 2 isoforms), NCX1, and InsP_3_R are complexed with ankyrin-B within a microdomain of cardiomyocyte T-tubules, and that the complex is deficient in ankyrin-B^+/−^ cardiomyocytes. Additionally, we present evidence that loss of the ankyrin-B-based complex is the molecular defect in cardiac arrhythmia due to ankyrin-B mutation in humans and mice. The ankyrin-B complex is not present in skeletal muscle, smooth muscle, or brain, and may have evolved in the context of specialized requirements for cytosolic Ca^2+^ regulation in cardiomyocytes.

## Results

### Ankyrin-B Is Required for T-Tubule Localization of NKA, NCX1, and InsP_3_R in Cardiomyocytes

**Figure 1:**
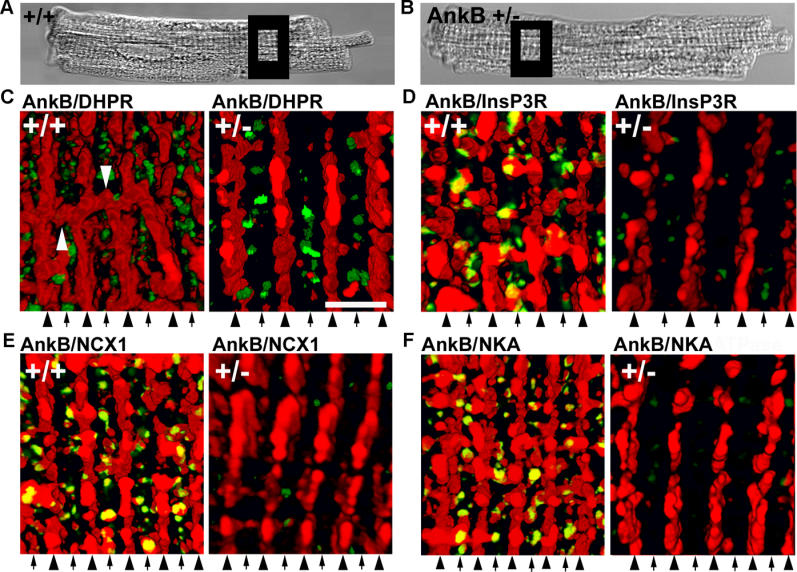
NCX1, NKA, and InsP_3_R Localization Require Ankyrin-B Boxes in differential interference contrast images (A and B) represent sites that were imaged in (C–F). Immunolocalization of ankyrin-B (red) and (C) DHPR (green), (D) InsP_3_R (green), (E) NCX1 (green), and (F) NKA (green) in wild-type (left) and ankyrin-B^+/−^ cells (right). Ankyrin-B^+/−^ myocytes were labeled and imaged using identical protocols. M-lines (middle of A-band) are denoted by arrowheads; T-tubules over I-bands are denoted by arrows. Scale bar = 2 μm. Distance between arrowheads is ~1.8 μm. Images represent at least twenty *Z*-sections at 0.18-μm intervals. In (C), perpendicular axial branches are denoted by white arrowheads.

Ankyrin-B as well as NKA, NCX1, and InsP_3_R are selectively lost from Z-line/T-tubule sites in haploinsufficient ankyrin-B^+/−^ cardiomyocytes [15]. The localization of these proteins was further resolved by three-dimensional rendering of consecutive confocal Z-sections of cardiomyocytes labeled by immunofluorescence (Figure 1). Wild-type ankyrin-B is organized in an intracellular tubular lattice in parallel with both the M-line and Z-line/T-tubules, but also including perpendicular axial branches that connect M-line and Z-line staining (Figure 1C). Z-line ankyrin-B staining is co-linear with the dyad marker DHPR in two-dimensional images, but is distinct from the DHPR in three dimensions (<2% of DHPR-positive voxels [three-dimensional (3D) pixels] overlap with ankyrin-B-positive voxels; not shown). Z-line ankyrin-B staining significantly overlaps in three dimensions with InsP_3_R (~45% of InsP_3_R-positive voxels co-localize with ankyrin-B-positive voxels; Figure 1D) as well as T-tubule-associated NCX1 (~53%; Figure 1E) and NKA (~51%; Figure 1F).

In contrast to wild-type cardiomyocytes, ankyrin-B^+/−^ cells lack Z-line staining as well as the axial lattice of ankyrin-B that connects Z-line- and M-line-associated populations (Figure 1C–1F, right panels). DHPR staining and organization is unaffected in ankyrin-B^+/−^ cardiomyocytes. InsP_3_R immunofluorescence (Figure 1D), as well as NCX1 and NKA isoform staining (Figure 1E and 1F) are markedly reduced at T-tubule sites. Residual InsP_3_R, NKA, and NCX1 rarely co-localize with ankyrin-B (levels of InsP_3_R, NKA, and NCX1 are reduced to levels too low for accurate determination of overlap).

### Ankyrin-B-Coupled NKA, NCX1, and InsP_3_R Are Co-Localized

**Figure 2:**
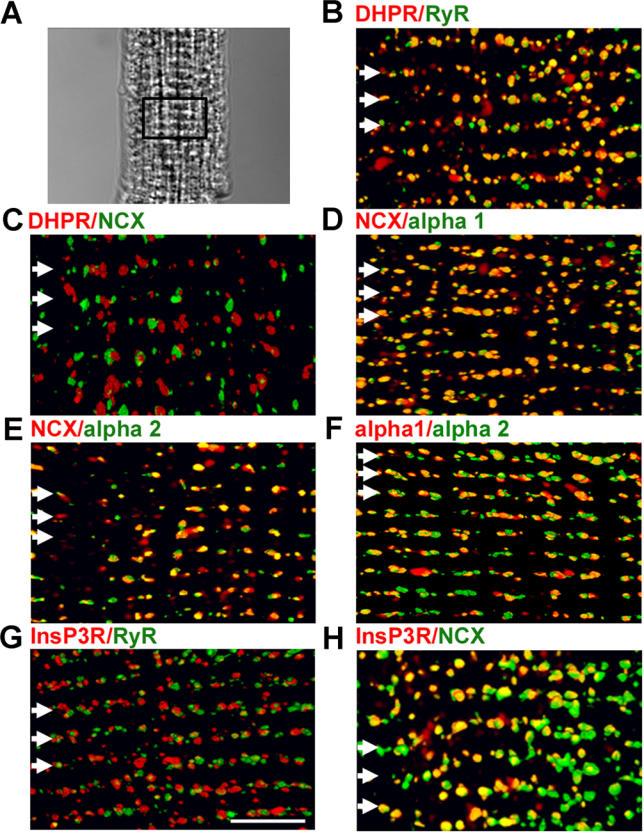
The Ankyrin-B-Based Complex of NKA, NCX1, and InsP_3_R Is Localized in a Specialized T-Tubule Microdomain of Cardiomyocytes Adult cardiomyocytes were labeled with indicated antibodies. 3D reconstructions of each cell (A, differential interference contrast) are shown for each combination. Staining pairs include (B) DHPR (red) and RyR (green), (C) DHPR (red) and NCX (green), (D) NCX (red) and NKA alpha1 (green), (E) NCX (red) and NKA alpha 2 (green), (F) NKA alpha 1 (red) and NKA alpha 2 (green), (G) InsP_3_R (red) and RyR (green), and (H) InsP_3_R (red) and NCX (green). Voxel co-localization was performed for non-sarcolemmal voxels. T-tubule/Z-lines are indicated by white arrows. Scale bar = 5 μm.

The Na/Ca exchanger has been localized at sites on cardiac T-tubules distinct from DHPR and RyR [19]. Moreover, NKA isoforms, NCX1, and InsP_3_R have been individually localized at T-tubule sites in cardiomyocytes [15,19–21]. However, localizations of all three proteins relative to each other have not been addressed. The relative localization of NCX1 with NKA alpha 1 and alpha 2 isoforms, InsP_3_R, DHPR, and RyR was evaluated using multiple combinations of double-labeled 3D images of mouse cardiomyocytes generated using confocal Z-stacks (Figure 2; Table 1). As expected, DHPR and RyR are co-localized, with ~55% voxel overlap of DHPR with RyR and ~42% overlap of RyR with DHPR (Figure 2B; Table 1), values comparable to voxel overlaps observed previously [19]. T-tubule NCX1 immunofluorescence (also localized at the sarcolemma [21]) is distinct from DHPR staining, with a voxel overlap of less than 5%, as reported previously [19] (Figure 2C; Table 1).

**Table 1:**
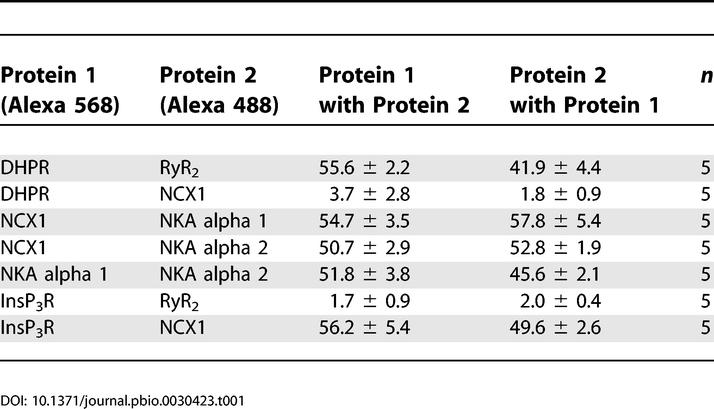
Analyses of the Extent of Voxel Co-Localization for Select Cardiac Protein Pairs

A new finding is that T-tubule NCX1 immunofluorescence co-localizes in submicron-sized domains with the T-tubule population of both NKA alpha 1 (Figure 2D; Table 1) and alpha 2 isoforms (Figure 2E; Table 1), with 55% (alpha 1) and 50% (alpha 2) voxel overlap. T-tubule NCX1 also co-localizes with InsP_3_R (Figure 2H; Table 1), with 56% of InsP_3_R voxels overlapping NCX1 and 50% of NCX1 voxels overlapping InsP_3_R. In contrast, InsP_3_R and RyR are not co-localized at T-tubule sites, with less than 5% voxel overlap (Figure 2G; Table 1). This is the first direct evidence to our knowledge that RyR and InsP_3_R are localized in spatially defined compartments of the endoplasmic reticulum and SR of ventricular cardiomyocytes.

NKA alpha 1 and alpha 2 isoforms were similarly distributed over the T-tubule (Figure 2F; Table 1) and sarcolemma (not shown). The T-tubule co-localization occurred in three dimensions as voxels with alpha 1 signal containing alpha 2 signal ~52% of the time, while voxels containing alpha 2 signal contained alpha 1 signal ~46% of the time. NKA isoforms have been proposed to have unique functions based on differences in localization and/or affinity for cardiac glycosides [22]. These differences may depend on the species and cell type, and have not been reported in mouse cardiomyocytes [23]. Our results in mouse ventricular cardiomyocytes suggest no major differences in NKA alpha 1 and alpha 2 localization at T-tubules (Figure 2) or sarcolemma (not shown). These results demonstrate co-clustering of NCX1, NKA, and InsP_3_R within microdomains along the T-tubule/SR that are distinct from classic T-tubule/SR junctions populated by DHPR and RyR. Moreover, the clusters of these proteins as well as ankyrin-B are reduced or absent in ankyrin-B^+/−^ cardiomyocytes.

### T-Tubule-Associated Ankyrin-B Is a Specialized Adaptation of Cardiomyocytes

**Figure 3:**
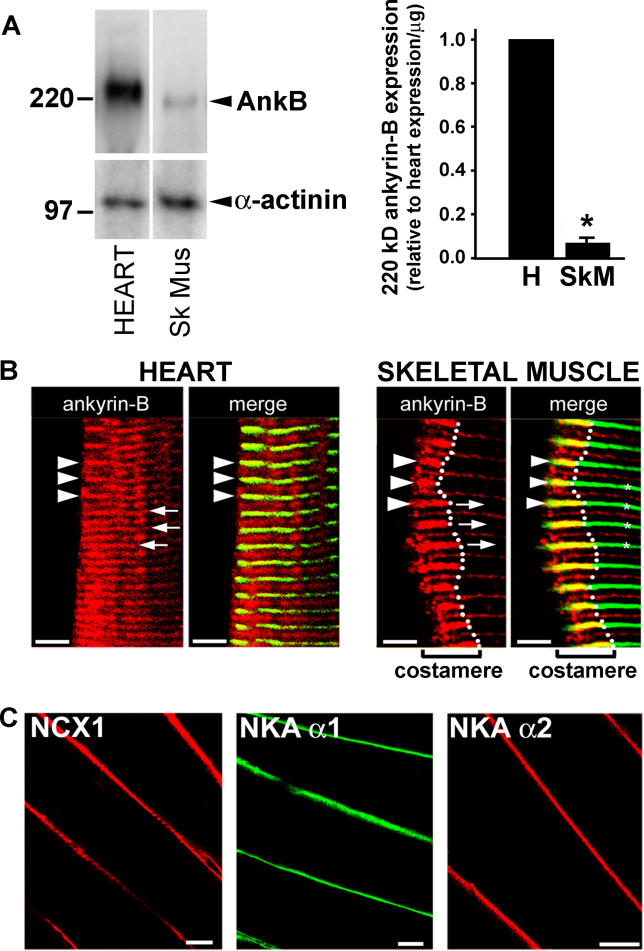
The Ankyrin-B-Based Complex Is a Specialized Feature of Heart (A) Relative expression of 220-kDa ankyrin-B in adult mouse heart and skeletal muscle (*n =* 3, *p <* 0.05). For the immunoblot in the left panel, 50 μg of total lysate was analyzed. (B) Ankyrin-B (AnkB) immunostaining (red) in frozen sections of heart (H) and skeletal muscle (Sk Mus, SkM). The Z-lines (arrowheads, also asterisks in right skeletal muscle panel) overlap with alpha-actinin (green). Arrows indicate A-bands. Ankyrin-B is localized at costameres in skeletal muscle, visualized as staining continuous with alpha-actinin near the sarcolemma, but does not extend into the interior. Ankyrin-B localizes over the A-band in both heart and skeletal muscle. Scale bars = 5 μm. (C) NCX1 (left panel), NKA alpha 1 (middle panel), and NKA alpha 2 immunostaining (right panel) in skeletal muscle. Scale bars = 18 μm.

The expression of 220-kDa ankyrin-B in skeletal muscle is nearly 10-fold lower than in heart (Figure 3A). Moreover, in contrast to ankyrin-B localization in cardiomyocytes (Figure 3B), ankyrin-B is not present over T-tubules of skeletal muscle, but instead is concentrated at punctate sites on the sarcolemma, over the A-band, and at costameres (Figure 3B). Additionally, in contrast to cardiac muscle, NCX1 and NKA isoforms are nearly undetectable over T-tubules of skeletal muscle, but are instead concentrated at the sarcolemma (Figure 3C). Finally, ankyrin-B expression is nearly absent from smooth muscle (Figure S1), and ankyrin-B-based complexes of NCX1, NKA isoforms, and InsP_3_R are not detectable in brain (see below). Therefore, the ankyrin-B-based complex of NKA, NCX1, and InsP_3_R is a specialized feature of cardiac myocytes.

### Ankyrin-B Coordinates NKA, NCX1, and InsP_3_R in a Macromolecular Protein Complex in Cardiomyocytes

**Figure 4:**
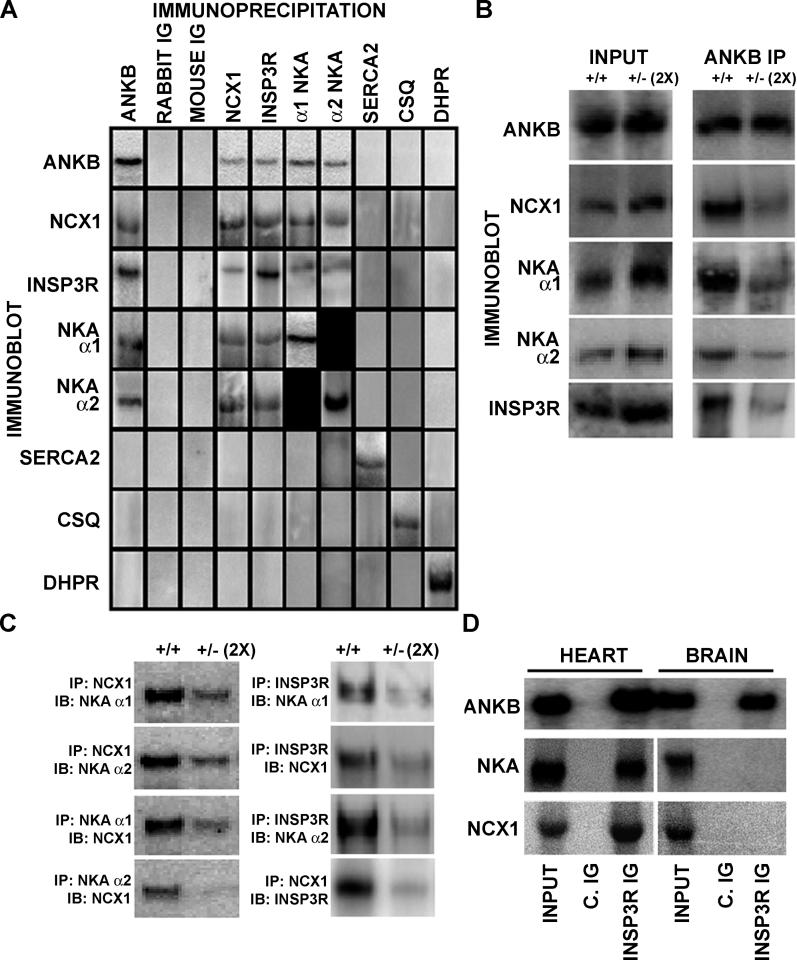
Ankyrin-B Forms a Macromolecular Complex with NKA, NCX1, and InsP_3_R That Is Missing in Ankyrin-B^+/−^ Heart (A) Immunoprecipitations and co-immunoprecipitations from detergent-soluble extracts from adult mouse heart. (B and C) Detergent-soluble lysates from wild-type or ankyrin-B^+/−^ mouse hearts were used for immunoprecipitations with indicated antibodies (IB, immunoblot; IP, immunoprecipitation). Immunoprecipitations of ankyrin-B^+/−^ extracts employed doubled amounts of input lysate to compensate for 50% reduction of ankyrin-B. (D) InsP_3_R co-immunoprecipitates 220-kDa ankyrin-B, NCX1, and NKA from detergent-soluble heart lysates (Input = 10%). In contrast, InsP_3_R co-immunoprecipitates 220-kDa ankyrin-B, but not NCX1 or NKA, from detergent-soluble lysates of mouse brain (Input = 10%). ANKB, ankyrin-B; C. IG, control Ig.

The finding that ankyrin-B is co-localized with NKA, NCX1, and InsP_3_R in cardiac T-tubule microdomains and that all of these proteins are coordinately reduced in ankyrin-B^+/−^ cardiomyocytes raises the question of their molecular organization. Given previous evidence that ankyrin-R can form heterocomplexes between two ankyrin-binding proteins [24], we wondered whether ankyrin-B could form a multi-protein complex involving NKA, NCX1, and InsP_3_R in cardiomyocytes. We therefore performed a series of immunoprecipitations from detergent extracts of mouse heart with antibodies against NCX1, alpha 1 and alpha 2 isoforms of NKA, and InsP_3_R, followed by immunoblots to detect associated proteins (Figure 4). As reported previously, ankyrin-B antibody co-immunoprecipitated NKA isoforms, NCX1, and InsP_3_R, but not DHPR, SR Ca^2+^ ATPase (SERCA2), or calsequestrin (Figure 4A) [15]. NCX1 antibody co-immunoprecipitated 220-kDa ankyrin-B as well as NKA alpha 1 and alpha 2 isoforms and InsP_3_R; DHPR, SERCA2, and calsequestrin were not co-immunoprecipitated (Figure 4). Moreover, NKA alpha 1– and alpha 2–specific antibodies co-immunoprecipitated 220-kDa ankyrin-B as well as NCX1 and InsP_3_R, but, again, not DHPR, SERCA2, or calsequestrin (Figure 4A). Finally, antibody specific for InsP_3_R also co-immunoprecipitated 220-kDa ankyrin-B along with NKA alpha 1 and alpha 2 and NCX1, but not DHPR, SERCA2, or calsequestrin. DHPR-, SERCA2-, and calsequestrin-specific antibodies did not co-immunoprecipitate 220-kDa ankyrin-B, NCX1, InsP_3_R, or NKA isoforms. These mutual co-immunoprecipitations provide evidence for a macromolecular protein complex in heart containing ankyrin-B coupled to alpha 1 and alpha 2 isoforms of NKA, NCX1, and InsP_3_R. While other proteins may be in this protein complex, components of the classic T-tubule/SR junction (DHPR [also RyR; not shown]) as well as components of the SR (SERCA2 and calsequestrin) are not included.

We next asked whether ankyrin-B was required for mutual co-immunoprecipitation of NKA, NCX1, and InsP_3_R by comparing wild-type hearts and ankyrin-B^+/−^ hearts, which are deficient in ankyrin-B (Figure 4B and 4C). Ankyrin-B^+/−^ hearts express reduced levels of 220-kDa ankyrin-B (decreased ~50%), NKA alpha 1 and alpha 2 (both reduced ~15%), NCX1 (reduced ~16%), and InsP_3_R (reduced ~33%) [15]. Strikingly, ankyrin-B^+/−^ heart lysates exhibited over 60% loss of the ability of ankyrin-B antibody to co-immunoprecipitate NKA, InsP_3_R, or NCX1, even when the quantity of lysate was doubled to equalize the starting amount of ankyrin-B (Figure 4B). Moreover, a similar reduction in NCX1 co-immunoprecipitation of Na/K pump isoforms and InsP_3_R occurred using doubled ankyrin-B^+/−^ lysates (Figure 4C). NKA alpha 1 and alpha 2 antibody also failed to co-immunoprecipitate a significant fraction of NCX1 or InsP_3_R from ankyrin-B^+/−^ doubled lysates. Finally, InsP_3_R antibody immunoprecipitated minimal levels of NCX1 or NKA isoforms from ankyrin-B^+/−^ heart (Figure 4C). These results demonstrate that a specialized population of ankyrin-B, which is reduced in ankyrin-B^+/−^ heart, is critical for ankyrin-B interactions with NKA alpha 1 and alpha 2, NCX1, and InsP_3_R.

Ankyrin-B, NKA, InsP_3_R, and NCX1 are all expressed in brain at levels comparable to those in heart tissue. However, while immunoprecipitation of 100,000*g* detergent extracts of brain tissue with antibody against the InsP_3_R co-immunoprecipitated ankyrin-B, NKA and NCX1 were not present (Figure 4D). Moreover, NKA and NCX1 were also missing when the immunoprecipitation was performed with antibody against ankyrin-B (not shown). These results are in contrast to the report that NCX1, NKA, InsP_3_R, and ankyrin-B co-immunoprecipitate along with several other proteins from 27,000*g* supernatants of detergent extracts from brain [25]. The difference could result from use of a 27,000*g* supernatant in the other study and a 100,000*g* supernatant in our experimental protocol. A major complication with a lower speed supernatant is the likely presence of large complexes such as those connected by short actin filaments that would be removed with more centrifugation. Our results demonstrate that co-expression of ankyrin-B with NCX1 and NKA in the same tissue is not sufficient for formation of a complex from a 100,000*g* supernatant.

### Reconstitution of an Ankyrin-B-Based Complex of NKA, NCX1, and InsP_3_R

**Figure 5:**
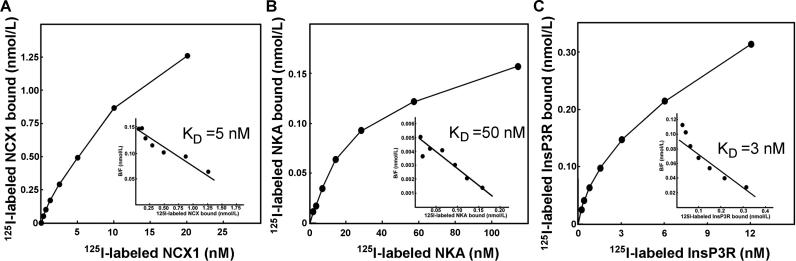
Purified NCX1, NKA, and InsP_3_R Directly Interact with Purified Ankyrin-B Membrane-Binding Domain Saturation binding of purified (A) ^125^I-labeled NCX1, (B) ^125^I-labeled NKA (NKA), and (C) ^125^I-labeled InsP_3_R with glutathione Sepharose–immobilized GST-ankyrin-B membrane-binding domain or GST control Sepharose. Inset for each panel represents Scatchard analysis of ankyrin-B interactions following subtraction of nonspecific binding to GST-Sepharose. Proteins were isolated as described in Materials and Methods.

Co-immunoprecipitation experiments as presented in Figure 4 provide evidence for interactions between ankyrin-B and its partners in vivo. We next evaluated whether ankyrin-B could form a complex with the NKA, NCX1, and InsP_3_R in vitro using purified proteins (See Materials and Methods). We first confirmed, using ^125^I-labeled proteins and immobilized ankyrin-B membrane-binding domain, that purified ankyrin-B membrane-binding domain directly interacts in vitro with purified NCX1 expressed in Sf9 cells (*K*_d_ = 5 nM), purified NKA from kidney (*K*_d_ = 50 nM), and purified InsP_3_R from cerebellum (*K*_d_ = 3 nM) (Figure 5).

**Figure 6:**
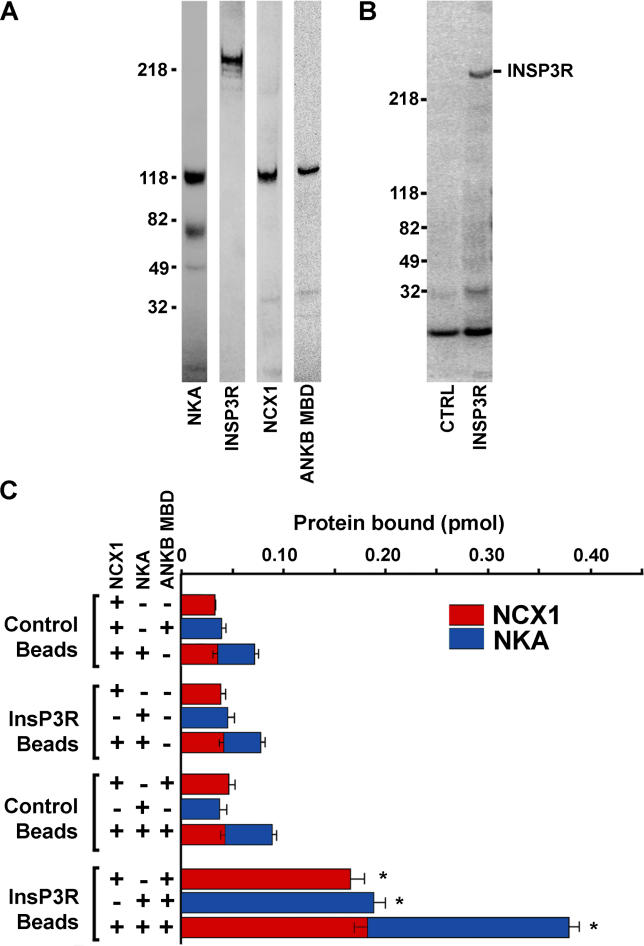
Reconstitution of the NCX1, NKA, and InsP_3_R Complex Requires Ankyrin-B (A) Purified NKA (α/β subunits), InsP_3_R, NCX1, and ankyrin-B membrane-binding domain (ANKB MBD) were examined by SDS-PAGE and Coomassie blue. (B) Stained gel of control beads and beads plus purified InsP_3_R. (C) NKA and NCX1 were ^125^I-labeled and incubated with control Dynabeads or Dynabeads coated with InsP_3_R with or without ankyrin-B membrane-binding domain. Bound protein was analyzed by a gamma-counter (C) and by SDS-PAGE and phosphorimaging (not shown; see Materials and Methods for protein measurement, *n =* 3, *p <* 0.05).

We next asked whether ankyrin-B could form a multivalent complex with these proteins. Association of InsP_3_R with NKA and NCX1 in the presence or absence of soluble ankyrin-B membrane-binding domain (purified as a GST-fusion protein and then cleaved from the GST-tag; Figure 6A) was assessed using biotinylated InsP_3_R bound to neutravidin-Dynabeads (Figure 6B) and ^125^I-labeled NCX1 and NKA. ^125^I-labeled NCX1 and ^125^I-labeled NKA associated with InsP_3_R-coated beads only in the presence of ankyrin-B membrane-binding domain (Figure 6C). In fact, while the intensity of the NCX1 band is ~50% of the band intensity of NKA (not shown), the picomoles of each protein bound to the InsP_3_R-coated beads was approximately equal (Figure 6C; specific activity of ^125^I-NKA ~504,000 cpm; ^125^I-NCX1 ~270,000 cpm). In contrast, ^125^I-labeled NCX1 and ^125^I-labeled NKA failed to bind to InsP_3_R-coated beads in the absence of ankyrin-B (Figure 6C). Additionally, in the presence of ankyrin-B, InsP_3_R-coated beads simultaneously associated with both ^125^I-labeled NCX1 and ^125^I-labeled NKA with no decrease in binding capacity compared to reactions where only one labeled protein was used (Figure 6C). These results demonstrate that interaction of InsP_3_R with either NCX1 or NKA is ankyrin-B-dependent and that these proteins can assemble in vitro in the absence of additional co-factors or regulatory proteins.

### Human E1425G Mutation Abolishes Ankyrin-B Association with NCX1, NKA, and InsP_3_R

**Figure 7:**
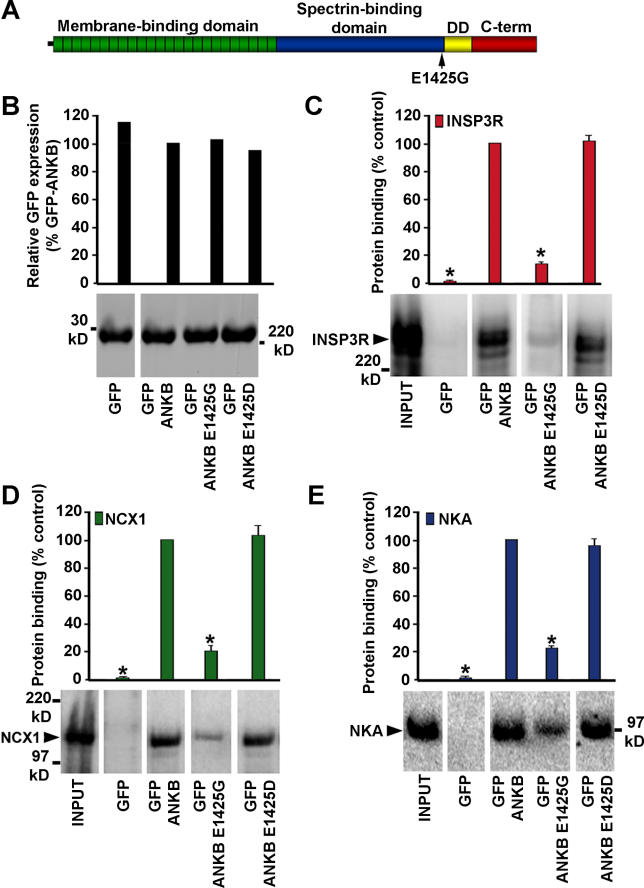
Human Ankyrin-B E1425G Mutation Abolishes Binding to NKA, NCX1, and InsP_3_R (A) 220-kDa ankyrin-B domain organization. The human LQT4 E1425G mutation is marked. (B) Immunoblot of expressed wild-type and mutant GFP-ankyrin-B proteins. (C–E) Relative binding of rat heart lysate (C) InsP_3_R, (D) NCX1, and (E) NKA to wild-type and mutant GFP-ankyrin-B proteins. Bound NCX1, NKA, and InsP_3_R were evaluated following quantitative immunoblot (pan-InsP_3_R, pan-NKA, and NCX1 Ig) and phosphorimaging (*n =* 3, *p <* 0.05).

One test of the physiological importance of the ankyrin-B-based complex is whether mutations in ankyrin-B resulting in loss of the complex also cause arrhythmia. E1425G mutation of ankyrin-B causes human cardiac arrhythmia and loss of activity of ankyrin-B in restoring normal Ca^2+^ waves to ankyrin-B^+/−^ neonatal cardiomyocytes [15]. The mechanism for loss of function due to the E1425G mutation, which is located close to the C-terminal regulatory domain and distant from the membrane-binding domain (Figure 7A), is not known. However, the regulatory domains of ankyrins (Figure 7A) can modulate activities of N-terminal membrane- and spectrin-binding domains [26,27].

The effect of the E1425G mutation on the ability of ankyrin-B to bind to NKA, NCX1, and InsP_3_R was evaluated using detergent extracts of heart tissue (not shown) and using purified proteins isolated as in Figure 6A. Evaluation of the binding properties of the E1425G mutant protein requires full-length 220-kDa ankyrin-B. We have not yet successfully generated full-length 220-kDa ankyrin-B in bacteria. Therefore, we used mammalian HEK293 cells to generate full-length wild-type and mutant ankyrin-B polypeptides for our binding studies. Recombinant green fluorescent protein (GFP)–220-kDa ankyrin-B that was either wild-type, with the E1425G mutation, or with a E1425D mutation was expressed and immuno-isolated from HEK293 cells using an affinity-purified antibody against GFP immobilized on Protein A agarose.

The levels of immobilized GFP-ankyrins were all equivalent in these assays (Figure 7B). E1425G ankyrin-B exhibited a 60%–70% loss of association with NCX1, NKA alpha 1 and alpha 2, and InsP_3_R from cardiac lysates (not shown), and as pure proteins (Figure 7C–7E). The conservative E1425D mutation at this site had no effect on binding of NCX1, NKA, or InsP_3_R (Figure 7C–7E). The finding that the E1425G mutation abolishes the ability of ankyrin-B to bind to NCX1, NKA, and InsP_3_R (Figure 7) suggests that interaction of ankyrin-B with either all three or some combination of these proteins is required for its function. It is possible that the E1425G mutation affects other protein interactions of ankyrin-B, although these remain to be identified.

## Discussion

This study presents the discovery of an ankyrin-B-based macromolecular complex of NKA (alpha 1 and alpha 2 isoforms), NCX1, and InsP_3_R in cardiomyocytes. The complex is localized in a microdomain along cardiomyocyte T-tubules resolved by 3D confocal microscopy as distinct from the classic dyad formed by DHPR and RyR. This microdomain was first described by Moore and colleagues, who also distinguished the T-tubule NCX1 from dyad proteins RyR and DHPR and from voltage-gated sodium channels by light microscopy using image deconvolution and wide-field epifluorescence microscopy [19]. T-tubule-associated ankyrin-B is a specialized adaptation of cardiomyocytes and is not evident in smooth muscle, which does not express significant ankyrin-B levels, or in skeletal muscle, where ankyrin-B is expressed at 10-fold lower levels than in heart. The T-tubule domain containing ankyrin-B-coupled NKA, NCX1, and InsP_3_R thus is a specialized adaptation of cardiac cells that is not present in other types of muscle cells.

**Figure 8:**
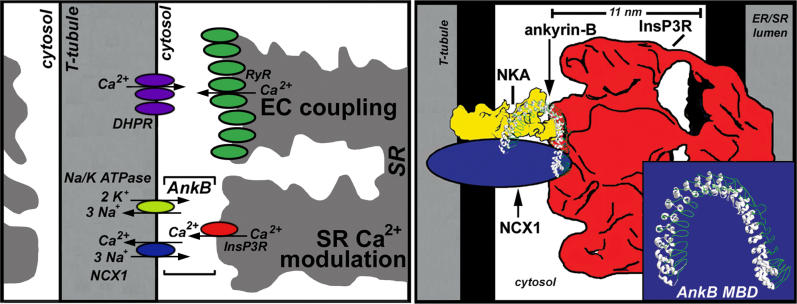
Model of the Cardiac Ankyrin-B Complex (Left) Model of ankyrin-B-dependent complex of NKA, NCX1, and InsP_3_R at T-tubule/SR sites distinct from the classic “dyad.” (Right) Scale model of ankyrin-B complex based on approximate dimensions of represented proteins. The protein ratios are not representative for in vivo couplings as there are likely 50–100 RyR_2_ for each InsP_3_R in a ventricular cardiomyocyte [32].

We propose a scale model for the ankyrin-B-based complex (Figure 8) based on previous structural reports and on evidence from this study that ankyrin-B can promote association between purified NKA, NCX1 and InsP_3_R (see Figure 6). In this scheme, the extended ankyrin-B membrane-binding domain adapts the NKA and NCX1 to the InsP_3_R in a configuration that would allow for regulation of cytosolic Ca^2+^ in a spatially privileged domain (Figure 8). It is likely that all participants in such an assembly have mutually interacting surfaces. In this case, the role of ankyrin-B could be to stabilize the assembly and/or possibly direct its cellular localization. The resulting macromolecular complex capable of coupled transport would accomplish the intended purpose of “restricted space” previously invoked to explain the action of cardiac glycosides [28]. However, the dimensions of a complex would be on the order of 10–20 nm, while an anatomical space or “synapse” between the endoplasmic reticulum and plasma membrane is 500–1,000 nm in size and would not provide a effective barrier to diffusion of small ions with radii less than 1 nm. A test of the idea of coupled transport by ankyrin-B-complexed proteins would be to selectively interfere with participation of individual members of the complex by knocking in mutants lacking ankyrin-B-binding activity.

While binding and localization data are consistent with simultaneous interaction of a single ankyrin-B molecule with NCX1, NKA, and InsP_3_ receptor, it also is possible that only one or two ankyrin-B-associated proteins are bound at a given time. It will be important in future experiments to isolate ankyrin-B-based macromolecular assemblies and directly determine stoichiometries of component proteins. A current challenge is that ankyrins also associate with spectrin and spectrin/actin complexes (reviewed in [17]), as well as proteins such as obscurin [29,30].

A role for InsP_3_R in heart is unknown. InsP_3_R-dependent Ca^2+^ signaling has been proposed to regulate excitation–contraction coupling in atrial myocytes by modulation of the activity (priming) of juxtaposed RyR [31]. However, based on the low ratio of InsP_3_R to RyR [32], the high Ca^2+^ buffering capacity of the cytosol [33], and now the distinct localizations of these Ca^2+^-release channels, it is unlikely that InsP_3_R Ca^2+^ release could affect the activity of RyR-mediated Ca^2+^-induced Ca^2+^ release in ventricular cardiomyocytes. Also, a role of InsP_3_R in Ca^2+^ signaling is difficult to reconcile with an environment where [Ca^2+^]_i_ transients occur continuously [2]. Our model suggests a counterintuitive role for InsP_3_R as a “Ca^2+^ pressure valve” for export of excess SR Ca^2+^ from the cell (Figure 8). Consistent with this idea is experimental evidence for functional coupling of SR Ca^2+^ stores with Ca^2+^ efflux [9,11].

Loss of the ankyrin-B-based complex may provide an explanation for the cardiac arrhythmia syndrome due to ankyrin-B mutations in humans and mice. The E1425G mutation of ankyrin-B, which causes human cardiac arrhythmia, also blocks binding of ankyrin-B to all three components (NCX1, NKA, and InsP_3_R) of the complex (see Figure 7). Moreover, ankyrin-B^+/−^ mice have a related cardiac arrhythmia, and ankyrin-B^+/−^ cardiomyocytes are also deficient in the complex, while the expression and subcellular localization of other cardiac ion channels and transporters (e.g., Na_v_ channels, which associate with a second ankyrin gene product, ankyrin-G) remain normal [15,18,34]. The electrical basis for ankyrin-B-dependent cardiac arrhythmia has been proposed, based on observations with ankyrin-B^+/−^ cardiomyocytes, to be due to elevated Ca^2+^ transients that provoke afterdepolarizations and extrasystoles following catecholamine-induced stress [15]. These predictions of a calcium-based phenotype are also supported by absence of abnormalities in the localization or expression of Na_v_ channels (also normal cardiac action potentials) and K channels in ankyrin-B^+/−^ and ankyrin-B^−/−^ cardiomyocytes [15,18]. Absence of the ankyrin-B complex would be predicted to result in less efficient export of calcium from the SR and could result in elevated calcium transients.

Ankyrin-B^+/−^ cardiomyocytes display preferential loss of ankyrin-B immunoreactivity at Z-line/T-tubule domains compared with M-line staining (see Figure 1). Potential explanations for this preferential loss may include reduced T-tubule ankyrin-B protein stability (half-life), increased T-tubule/SR membrane turnover, or differences in the association of each ankyrin-B population with the underlying cytoskeleton. Alternatively, reduced expression of the T-tubule/SR population of ankyrin-B in ankyrin-B^+/−^ cardiomyocytes may result from differences in the molecular identities of ankyrin-B polypeptides at each domain. For example, ankyrin-B immunoreactivity at the M-line may represent an ankyrin-B splice form that lacks *Ank2* exon 23 (exon targeted in the ankyrin-B knock-out mouse) but still reacts with ankyrin-B Ig.

Interaction between InsP_3_R and ouabain-associated Na/K pump has been reported to be responsible for slow Ca^2+^ oscillations in cultured renal proximal tubule and Cos7 cells [35]. Our results with pure proteins suggest that InsP_3_R and the NKA do not interact directly, at least not with high affinity (see Figure 7). Thus, it will be of interest to evaluate possible participation of ankyrin-B or possibly other adaptor proteins in this system. More generally, determinants of cellular localization and partnerships with physiologically related proteins likely are an essential aspect of function for all ion channels and transporters. Ankyrins are ubiquitously expressed and display diversity in protein interactions. Based on the findings of this study, and previous findings that ankyrin-G is required for coordinating voltage-gated Na channels and L1CAM cell adhesion molecules at axon initial segments [36,37], we predict that ankyrins are likely to contribute to higher order organization of multiple channels and transporters in a variety of tissues.

## Materials and Methods

### Animals

Mice used in these studies were adult WT C57BL/6 mice and ankyrin-B^+/−^ littermates (C57BL/6), 3–6 mo of age and weighing 30–40 g. Animals were handled according to approved protocols and animal welfare regulations of the Institutional Review Board of Duke University Medical Center. Mouse ventricular cardiomyocytes were isolated as described in [15].

### Immunofluorescence

Antibodies not described in [15] include NCX1 (Affinity Bioreagents, Golden, Colorado, United States; Swant, Bellinzona, Switzerland), alpha 1 and alpha 2 ATPase (Transduction), DHPR (ABR, Alomone), affinity-purified GFP polyclonal Ig, and affinity-purified pan-InsP_3_R polyclonal Ig generated against the C-terminus of mouse InsP_3_R (residues 2592 −2750). When unavoidable, mouse cells were immunostained with monoclonal antibodies that had been first affinity-purified. For these monoclonal antibodies, we confirmed that our staining was specific by control experiments in rat cells. Additionally, Alexa anti-mouse secondary antibodies were examined for background immunoreactivity. Adult cardiomyocytes were stained as described [15]. Isolated ventricular mouse cardiomyocytes were double-labeled and imaged in three dimensions by rendering of confocal *Z*-scans obtained at 0.18-μm increments near the center of isolated cells using a 100 power/1.45 NA objective (LSM 510, Zeiss, Oberkochen, Germany). LSM *Z*-stacks were transferred to Volocity software (Improvision, Lexington, Massachusetts, United States), and identical protocols were used for 3D rendering of WT and ankyrin-B^+/−^ cells. Volocity Classification software or LSM 510 software was used to measure voxel or pixel co-localization. Data represent at least three separate experiments with at least five areas measured for each experiment. Areas measured do not include sarcolemmal membrane voxels. Using monoclonal and polyclonal antibody directed against the same protein, cardiac double-labeling, and voxel co-localization revealed that the maximal co-localization for the same protein was ~65% consistent with previous studies [19].

### Ankyrin-B mutagenesis***.***

GFP–220-kDa ankyrin-B mutants E1425G and E1425D were created using site-directed mutagenesis. The mutated region was subcloned into a native GFP–220-kDa ankyrin-B plasmid, and the plasmid was completely sequenced to verify that no additional mutations were introduced.

### Statistics

When appropriate, data were analyzed using a two-tailed Student's *t*-test, and values less than *p <* 0.05 were considered significant. Values are expressed as the mean ± standard deviation.

### Protein modeling

3D protein structures for the model in Figure 8 were approximated based on published structures [38–43].

### Immunoprecipitation and solubilization of heart proteins

Adult heart immunoprecipitations and quantitative immunoblotting were performed as described [15]. Briefly, adult mouse heart and brain were dissected and rinsed in PBS plus 0.32 M sucrose and 2 mM Na EDTA, flash frozen in liquid nitrogen, and ground into a fine powder. The powder was resuspended in 4 volumes of 50 mM Tris HCl (pH 7.35), 10 mM NaCl, 0.32 M sucrose, 5 mM Na EDTA, 2.5 mM Na EGTA, 1 mM PMSF, 1 mM 4-(2-aminoethyl) benzenesulfonylfluoride hydrochloride (AEBSF), 10 μg/ml leupeptin, and 10 μg/ml pepstatin using a Dounce homogenizer (Kimble/Kontes, Vineland, New Jersey, United States). The homogenate was centrifuged at 1,000*g* to remove nuclei. Triton X-100 and deoxycholate were added to the post-nuclear supernatant for final concentrations of 1.5% Triton X-100 and 0.75% deoxycholate. The lysate was pelleted at 100,000*g* for 1 h at 4 °C, and the supernatant was re-cleared at 100,000*g* for 1 h to remove residual large membranes or vesicles. The resulting supernatant was used for immunoprecipitation (see Figure 4) as described [16], or for binding experiments.

### Binding studies

GFP–220-kDa ankyrin-B and mutants (E1425G and E1425D) were expressed in HEK293 cells and purified using affinity-purified GFP Ig coupled to Protein A agarose beads. Briefly, cells were lysed in above homogenization buffer plus 1.0% Triton X-100 and 0.5% deoxycholate. The extract was centrifuged at 100,000*g,* and the supernatant was incubated with GFP affinity-purified Ig coupled to Protein A sepharose. The beads were washed with homogenization buffer plus 1.0% Triton X-100. Purified proteins were incubated with 10 μg of affinity-purified GFP Ig or control Ig coupled to Protein A sepharose beads for 4 h at 4 °C. The beads were washed four times with homogenization buffer plus 1.0% Triton X-100. Protein bound to each mutant GFP–220-kDa ankyrin-B was eluted, analyzed by quantitative ^125^I-labeled Protein A immunoblot, normalized for relative GFP–ankyrin-B expression, and then compared to WT GFP–220-kDa ankyrin-B binding.

For in vitro complex reconstitution experiments, 0.5 ml of Dynabeads M-270 Epoxy (1 × 10^9^) beads (Dynal Biotech, Brown Deer, Wisconsin, United States) were washed in PBS and incubated with 5 mg of neutravidin in PBS in a final volume of 1 ml for 48 h at 25 °C. Beads were then washed in BSA binding buffer (20 mM Hepes [pH 7.3], 50 mM NaCl, 1 mM Na EDTA, 0.1% Triton X-100, 1 mM sodium azide, and 5 mg/ml BSA). Purified InsP_3_R (0.5 ml; 200 μg/ml) was incubated with a 20-fold molar excess of NHS-LC-biotin (Pierce Biotechnology, Rockford, Illinois, United States) overnight at 4 °C. For control biotin, the same biotin was incubated overnight in PBS. Biotin-InsP_3_R was then dialyzed against binding buffer without BSA to remove unbound biotin. Then 50% of the neutravidin Dynabeads were pre-incubated with the control biotin for 2 h at 4 °C, washed, and resuspended in BSA binding buffer (control neutravidin Dynabeads). Neutravidin Dynabeads were incubated with the dialyzed biotin-InsP_3_R while control neutravidin Dynabeads were incubated with the same concentration of unlabeled InsP_3_R for 2 h at 4 °C. Dynabeads were then washed 3× in BSA binding buffer and used for binding experiments. Coated Dynabeads (InsP_3_R at 20 nM) were incubated with ^125^I-labeled NCX1 (20 nM final concentration; specific activity 274,000 cpm/pmol) and/or ^125^I-labeled NKA (20 nM final concentration; specific activity ~504,000 cpm/pmol) in the presence or absence of a pre-incubation with ankyrin-B membrane-binding domain (20 nM final concentration) in a final volume of 50 μl of BSA binding buffer (20 mM Hepes [pH 7.3]; 50 mM NaCl, 1 mM Na EDTA, 0.2% Triton X-100, 1 mM NaN_3_, and 5 mg/ml BSA). Following 4 h, the beads were washed in binding buffer minus BSA, and both pellet and supernatant samples were assayed for ^125^I in a gamma counter (*n =* 3). The samples were then examined by SDS-PAGE and phosphorimaging (*n =* 3). Values for picomoles bound of ^125^I-labeled NCX1 or ^125^I-labeled NKA in experiments where only one labeled ligand was used were calculated from counts of ^125^I-labeled protein bound and specific activity. Values for picomoles bound of ^125^I-labeled NCX1 and ^125^I-labeled NKA when two labeled proteins were incubated together (^125^I-labeled NCX1 + ^125^I-labeled NKA) were calculated by first determining a ratio of counts of ^125^I-labeled protein bound/band intensity for unique bands of ^125^I-labeled NCX or ^125^I-labeled NKA in single protein binding experiments (i.e., ^125^I-labeled NCX + ankyrin-B + InsP_3_R beads). The intensity of these same bands was measured in the gel lanes where the two proteins (i.e., ^125^I-labeled NCX + ^125^I-labeled NKA + ankyrin-B + InsP_3_R beads) were combined to determine the number of picomoles bound of each protein. We observed approximately equal picomoles of ^125^I-labeled NCX1 and ^125^I-labeled NKA bound to InsP_3_R beads when ankyrin-B was included in the binding reaction. However, because of the lower specific activity of ^125^I-labeled NCX1, the band intensity on the gel was approximately 50% of that of ^125^I-labeled NKA. Saturation binding was performed essentially as described in [16] but using glutathione beads. Briefly, ankyrin-B membrane-binding domain was purified as described in [16]. Increasing concentrations of ^125^I-labeled InsP_3_R, ^125^I-labeled NCX1, or ^125^I-labeled NKA were incubated for 2 h at 25 °C with glutathione Sepharose-immobilized GST-ankyrin-B membrane-binding domain or GST. The beads were washed and counted in a gamma counter. The data were corrected for nonspecific binding at each concentration by subtracting values obtained with GST-coated beads.

### Protein purification

Full-length human NCX1 was cloned from a human heart library (Clontech, Palo Alto, California, United States) into pBacPak9 (Clontech) using standard molecular techniques. For purification of NCX1, a His-tag was engineered to the C-terminus. NCX1 was expressed in SF21 insect cells using a generated recombinant baculovirus. Cells were infected in monolayer cultures with a MOI of ten for 72 h at 27 °C. Cells were harvested and washed in PBS, and cell pellets were snap frozen and stored at −80 °C. All subsequent procedures were performed at 4 °C in the presence of protease inhibitors (100 μg/ml AEBSF, 100 μg/ml benzamidine, 30 μg/ml leupeptin, and 10 μg/ml pepstatin). Cells were syringed and sonicated in cell homogenization buffer (PBS, 1 mM Na EDTA, 1 mM DTT, and 1 mM sodium azide) to break the cell membranes, then centrifuged at 100,000*g* for 30 min to collect membranes. Cell membranes were pre-extracted with 20 mM CHAPS (pH 12) for 30 min followed by 20 mM PB (pH 7.3), 0.5 M NaCl, 0.5 M urea, 0.5% Triton X-100, and 0.5 mM beta mercaptoethanol. The cell residue was resuspended in extraction buffer (50 mM PB (pH 8.0), 0.3 M NaCl, 10 mM imidazole, 0.2% Triton X-100, 1 mM beta mercaptoethanol, 1 mM sodium azide, and 2% Sarkosyl) for 20 min. The extract was centrifuged at 100,000*g* for 1 h, and the supernatant collected and diluted 10-fold in buffer lacking Sarkosyl. The diluted extract was applied to a column of Ni-NTA Sepharose, washed with 10–20 column volumes of dilution buffer, and eluted with buffer plus 0.3 M imidazole. Peak fractions were pooled, adjusted to 10% glycerol, snap frozen, and stored at −80 °C. Sheep kidney NKA was isolated in membrane-bound form from outer medulla as previously described [44]. The NKA was extracted and purified as previously described [45]. The InsP_3_R was purified from frozen bovine brain cerebellum by a modification of published procedures [16,46]. All procedures were carried out at 4 °C in the presence of protease inhibitors (100 μg/ml AEBSF, 100 μg/ml benzamidine, 30 μg/ml leupeptin, and 10 μg/ml pepstatin). Cerebellum was homogenized using a polytron in five volumes (weight/volume) of homogenization buffer (10 mM Hepes [pH 7.3], 0.32 M sucrose, 2 mM EGTA, 1 mm DTT, and 1 mM sodium azide), and centrifuged at 2,000 rpm for 10 min. Membranes were then collected at 30,000*g* for 1 h. Membranes were prewashed in wash buffer (50 mM Tris HCl [pH 8.0], 1 mM Na EGTA, 1 mM DTT, and 1 mM Na azide), then resuspended to the homogenization volume with the wash buffer. InsP_3_R was extracted from the membranes by the addition of 2% final Triton X-100 for 30 min, and supernatants collected after centrifugation at 30,000*g* for 1 h. The extract was adjusted to 0.25 M NaCl and applied to a 50-ml heparin Sepharose column equilibrated in 0.25 M NaCl and 0.2% Triton X-100 extraction buffer. The heparin Sepharose was washed with ten column volumes of equilibration buffer, and then eluted with 0.5 M NaCl buffer. Peak fractions were pooled and dialyzed against ten volumes of column buffer lacking NaCl and 20 mM Tris HCl (pH 8.0). A precipitate formed after dialysis and was collected by centrifugation at 100,000*g* for 20 min. The pellet was resuspended in column buffer with the addition of 1.0 M NaCl and was re-centrifuged as above. The InsP_3_R released into the supernatant was then adjusted to 0.2 mM CaCl_2_ and 0.2 mM MnCl_2,_ and applied to a 4-ml ConA Sepharose column. The column was washed in 20 column volumes of buffer, the elution started with the addition of 1 M mannose, the elution stopped, and the column allowed to sit in elution buffer overnight. The elution was continued the following day and fractions collected, aliquoted, snap frozen, and stored at −80 °C.

## Supporting Information

Figure S1 Ankyrin-B-Based Complex Is a Specialized Feature of Cardiac Myocytes Ankyrin-B is expressed in ventricular cardiomyocytes but not in smooth muscle lining large arteries. Image represents adult mouse heart immunostained with ankyrin-B-specific Ig.
(2.7 MB TIF).

### Accession Number

The NCBI (http://www.ncbi.nlm.nih.gov/) accession number for ankyrin-B is NM_020977.

## Abbreviations

3D: three-dimensional
AEBSF: 4-(2-aminoethyl) benzenesulfonylfluoride hydrochloride
DHPR: dihydropyridine receptor
GFP: green fluorescent protein
InsP_3_R: inositol 1,4,5-trisphosphate receptor
NCX1: Na/Ca exchanger 1
NKA: Na/K ATPase
RyR: ryanodine receptor
SERCA2: sarcoplasmic reticulum Ca^2+^ ATPase
SR: sarcoplasmic reticulum
T-tubule: transverse tubule
